# Synthesis of amorphous and graphitized porous nitrogen-doped carbon spheres as oxygen reduction reaction catalysts

**DOI:** 10.3762/bjnano.11.1

**Published:** 2020-01-02

**Authors:** Maximilian Wassner, Markus Eckardt, Andreas Reyer, Thomas Diemant, Michael S Elsaesser, R Jürgen Behm, Nicola Hüsing

**Affiliations:** 1 Chemistry and Physics of Materials, Salzburg University, A-5020 Salzburg, Austria; 2 Institute of Surface Chemistry and Catalysis, Ulm University, D-89069 Ulm, Germany

**Keywords:** amorphous carbon, graphitized carbon, hydrothermal carbonization, nitridation, nitrogen doping, oxygen reduction reaction (ORR), porosity

## Abstract

Amorphous and graphitized nitrogen-doped (N-doped) carbon spheres are investigated as structurally well-defined model systems to gain a deeper understanding of the relationship between synthesis, structure, and their activity in the oxygen reduction reaction (ORR). N-doped carbon spheres were synthesized by hydrothermal treatment of a glucose solution yielding carbon spheres with sizes of 330 ± 50 nm, followed by nitrogen doping via heat treatment in ammonia atmosphere. The influence of a) varying the nitrogen doping temperature (550–1000 °C) and b) of a catalytic graphitization prior to nitrogen doping on the carbon sphere morphology, structure, elemental composition, N bonding configuration as well as porosity is investigated in detail. For the N-doped carbon spheres, the maximum nitrogen content was found at a doping temperature of 700 °C, with a decrease of the N content for higher temperatures. The overall nitrogen content of the graphitized N-doped carbon spheres is lower than that of the amorphous carbon spheres, however, also the microporosity decreases strongly with graphitization. Comparison with the electrocatalytic behavior in the ORR shows that in addition to the N-doping, the microporosity of the materials is critical for an efficient ORR.

## Introduction

Fuel cells and metal–air batteries are important renewable energy technologies. Both rely on the oxygen reduction reaction (ORR). The best established ORR catalysts are so far based on Pt nanoparticles or Pt alloys. However, Pt is expensive and its stability under fuel-cell working conditions is limited. Therefore, alternative catalysts based on noble-metal-free, less expensive and stable materials are highly needed. Metal-free carbon materials, single- or multi-doped with N, B, P, S, halogens, Si or Se, have turned out to be promising ORR catalysts [1–6]. N-doped carbon materials show promising ORR activities along with high electric conductivity, in addition they can result in further advantages such as an improved tolerance towards impurities compared to Pt-based catalysts [1]. A wide variety of N-doped carbon materials is known from the literature, reaching from N-doped graphene and graphite, N-doped carbon nanotubes, carbon cages, carbon cups and carbon fibers [7–10], N-doped 3D ordered (meso)porous carbon materials [11], N-doped carbon composites (e.g., carbon nanotubes/graphene) [12], and N-doped carbon spheres [13–14] to graphitic-C_3_N_4_ carbon nitride composites [15].

In the present work we report results of a systematic study on the synthesis and characterization of N-doped carbon spheres as possible ORR catalysts. Before presenting our results, we will briefly summarize relevant previous findings. There are two main strategies for the synthesis of N-doped carbons: first, in situ doping with nitrogen, employing C- and N-containing precursors directly in the synthesis of the material, which leads to the direct formation of C–N bonds; and second, post-synthesis N-doping via substitutional incorporation of N into the carbon lattice of as-synthesized carbon materials with a reactive nitrogen-containing agent. Established in situ syntheses are chemical vapor deposition (CVD) and arc discharge methods for N-doped graphene, graphite, and carbon nanotubes [9]. Most commonly, the post-synthetic approach is carried out by thermal treatment of carbon in ammonia atmosphere, typically leading to surface N-doping. A variety of N bonding configurations can be formed within the carbon lattice [8], among them the pyridinic and quarternary (also: graphitic) nitrogen bonding configurations were assumed as origin of the ORR activity of these N-doped carbon materials [16]. The exact nature of the active site is controversially discussed; some researchers ascribe the ORR activity to graphitic nitrogen sites [17–21], while others propose pyridinic nitrogen as more important [16,22–26]. Previous results of our groups indicate that the ORR activity of nitrided carbon is dominated by the carbon edge atoms of micropores in graphenic structures and the electronic structure of those atoms which is additionally modified by low-level N-doping [26–27]. This may include both graphitic and pyridinic N-doping. Kim et al. [28] suggested that both bonding situations interconvert during the ORR and that both might be equally important. A directed tailoring of the active sites in the carbon material is a prerequisite for a knowledge-based optimization of the ORR activity. As reported by Lai et al. [18], this can be achieved to a certain extent by varying the reaction temperature and the utilization of different N(C) precursors. Annealing graphene oxide (GO) in an ammonia atmosphere at 550 °C led to pyridinic N-doped graphene, while at a temperature of 850 °C graphitic nitrogen coexisted with pyridinic nitrogen, and for higher temperatures the amount of graphitic N increased. Annealing GO at 850 °C in the presence of polyaniline or polypyrrole instead of ammonia resulted in pyridinic or pyrrolic N moieties, respectively [18].

Beside the N bonding configuration, the ORR activity is affected by the N content, the surface area (porosity) and possibly the degree of graphitization [27]. The nitrogen content defines, among others, the density of N sites, which influence the ORR activity even if they are not the active ORR sites themselves [27]. The N content depends on the amount of nitrogen in the precursor, the N(C) precursor concentration, the reaction temperature as well as the duration of the doping treatment. During ammonia nitriding of carbon a significant increase of the N content occurs at reaction temperatures higher than 550 °C (formation of nitriding-active species based on ammonia decomposition), and at ca. 650 °C the maximum N content is reached. For higher temperatures, the N content decreases again, here the formation of C–C bonds is favored over the formation of C–N bonds. At a given reaction temperature, the N content increases with higher ammonia concentrations up to a certain maximum, however, only a limited amount of N can be incorporated. Exceeding this point leads to the formation of defects causing decomposition of the carbon framework and a decrease of the N content [29–30]. The availability of active sites (for a certain electrochemical reaction) correlates with the electrochemically active surface area for this reaction. For most conventional porous carbon materials micropores contribute significantly to the surface area, but their small pore size is considered to only allow a limited mass transport, which might result in a low accessibility of the active sites therein for electrochemical processes. Investigations of N-doped 3D ordered porous carbon materials showed, e.g., that a high content of meso- and macropores is beneficial for the ORR activity [11]. Finally, a higher degree of graphitization generally leads to an improved electrical conductivity, which should improve the overall ORR activity. On the other hand, it may alter also the properties of the active sites, which may be either beneficial or disadvantageous. The degree of graphitization can be increased, e.g., by higher reaction temperatures or catalytic graphitization [31–33].

Previously, we had reported on core–shell titanium (oxy)nitride and tantalum (oxy)nitride@N-doped carbon composite spheres, which were based on a similar conducting carbon core as investigated in the present study, and their performance as ORR catalysts [34–35]. For these systems, which turned out to be promising ORR catalysts, we found that nitriding the metal-oxide shell concomitantly results in drastic structural changes and nitriding of the carbon core. In this study we aim at gaining deeper insights in the role of the N-doped spherical carbon core in the ORR. We are well aware of the fact that many groups reported the synthesis of N-doped carbon spheres and some even their application in electrocatalysis, however, a fundamental and detailed discussion of the changes during nitridation regarding chemistry, structure and morphology combined with a correlation of the ORR activity for these materials has been, to our knowledge, not provided. In this study we also added a discussion on the influence of a higher degree of graphitization on the ORR by comparison of as-synthesized and pre-graphitized N-doped carbon spheres. We believe that the use of structurally and chemically well-defined model systems, which necessarily might not be as sophisticated as other reported materials, is the proper way to gain a fundamental understanding of correlations between structural properties and electrochemical performance.

In the following we will first give a detailed structural and chemical analysis of the resulting materials (section 1 in “Results and Discussion”), followed by a discussion of their performance as ORR catalyst in electrocatalytic measurements under controlled electrolyte transport, employing a rotating ring disk electrode (RRDE) setup (section 2 in “Results and Discussion”). We will compare the ORR performance with those of the previously reported TiON@NCS and TaON@NCS composite materials. A more detailed account of the electrochemical properties and of the ORR activity and mechanism is given elsewhere [27].

## Results and Discussion

### 1 Synthesis and structural/chemical analysis of (graphitized) N-doped carbon spheres

**Figure 1:**
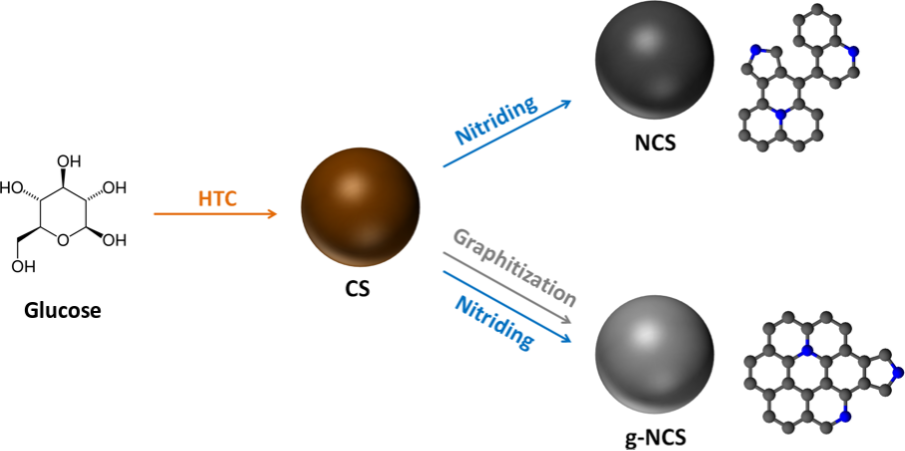
Schematic synthesis overview of amorphous N-doped carbon spheres (NCSs) and graphitized N-doped carbon spheres (g-NCSs) by hydrothermal carbonization (HTC) of a glucose solution yielding carbon spheres that are either directly nitrided with ammonia or graphitized and then nitrided (nitrogen atoms in the graphitic lattice are given in blue).

Carbon spheres were synthesized by hydrothermal treatment of a glucose solution (Figure 1), following a previously reported approach [36]. They were either directly nitrided (nitrided carbon spheres, NCSs) or graphitized and then nitrided (graphitized nitrided carbon spheres, g-NCSs). Heat treatments, which are necessary for the nitridation but also for the graphitization, were performed between 550 and 1000 °C (with steps of 150 °C).

**Figure 2:**
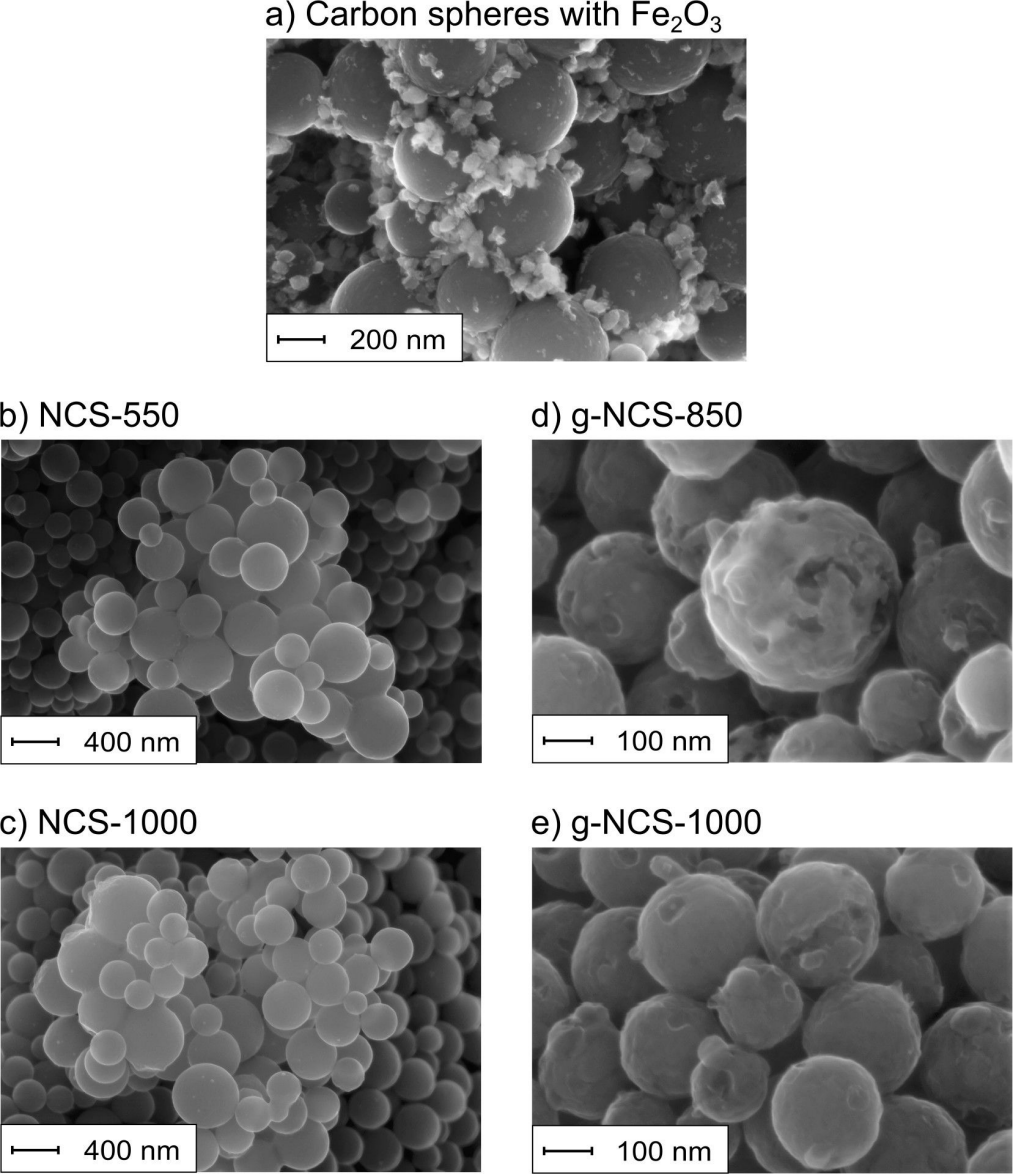
SEM images of (a) the carbon spheres with Fe_2_O_3_ before acid treatment, (b, c) the different non-graphitized and (d, e) the graphitized carbon spheres.

The as-synthesized carbon particles show a well-defined spherical shape with diameters of 330 ± 50 nm and a smooth surface (see also the scanning electron microscopy (SEM) image in Figure 2a). Fe_2_O_3_ particles, as graphitization catalyst, are loaded successfully on pre-carbonized carbon spheres; yet there are domains of higher or lower loadings. After nitriding with ammonia, g-NCS-550, g-NCS-700 and all samples of the NCS series still show a spherical shape with a smooth surface (Figure 2b and Figure 2c show, respectively, NCS-550 and NCS-1000 as examples). No remaining catalyst particles are observed for the graphitized samples via energy dispersive X-ray spectroscopy (EDX) and X-ray photoelectron spectroscopy (XPS). However, we cannot fully exclude small amounts of residual iron in the graphitized catalysts below the detection limit of XPS (about 0.2 atom %) and EDX (about 0.1 wt %).

**Figure 3:**
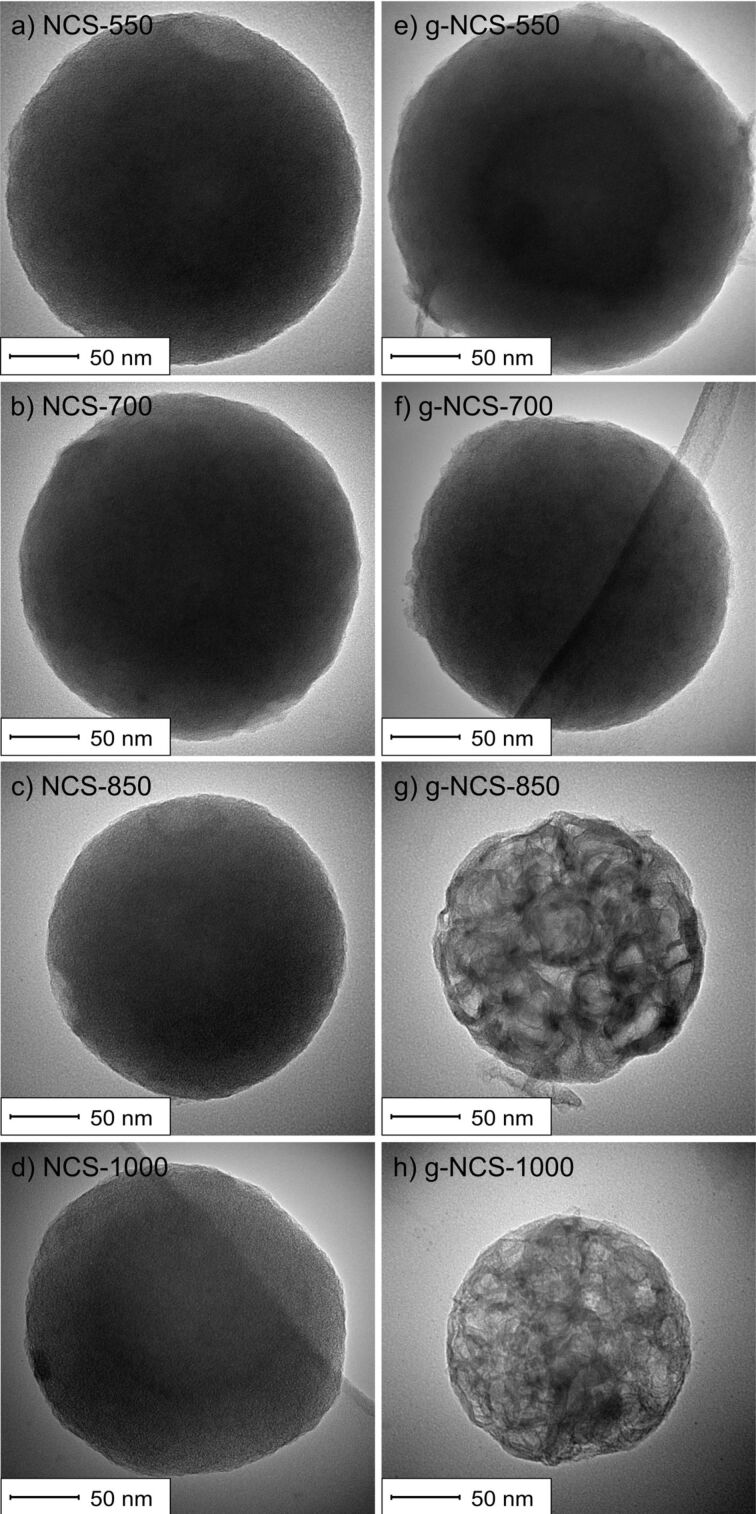
TEM images of (a–d) the NCS catalysts and (e–h) the g-NCS catalysts. The TEM images in (a–d) are reprinted with permission from [27], copyright 2019 Elsevier.

**Figure 4:**
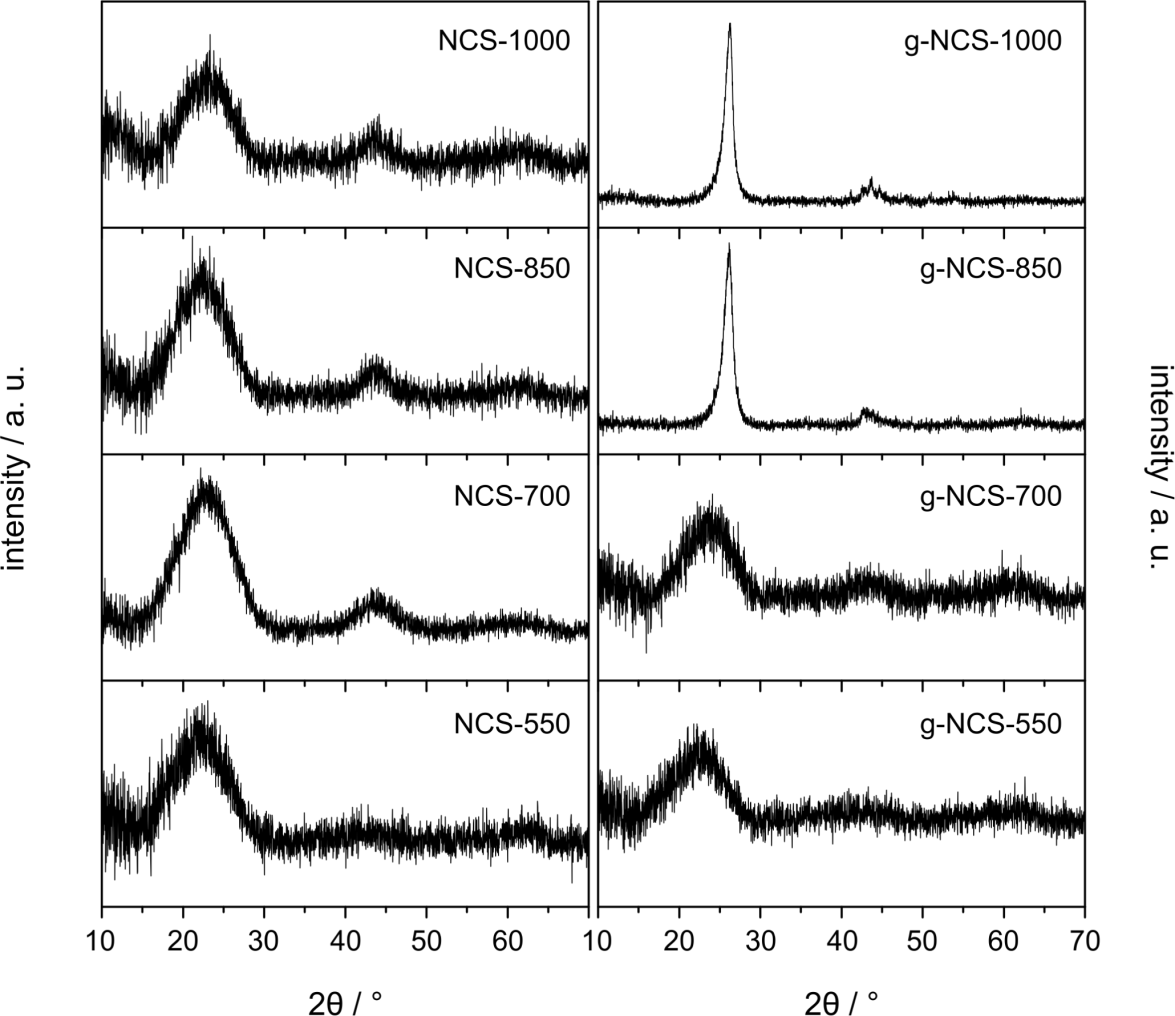
X-ray diffraction patterns of the NCS and g-NCS catalyst series.

TEM images (Figure 3a–d) reveal no highly ordered domains (e.g., graphene layers) of the said N-doped carbon spheres, which is in good agreement with the results of the X-ray diffraction measurements (XRD, Figure 4), confirming an amorphous carbon structure for all particles mentioned so far. Upon catalytic graphitization of g-NCS-850 and g-NCS-1000, the smooth surface becomes texturized or perforated as seen in the SEM images (cf. Figure 2d,e), and the spheres partially erode. This can be explained as a result of catalytic graphitization, for which the following mechanism was proposed by Nettelroth et al. [32]: The catalyst particles carve themselves into the underlying carbon atom structure by a redox reaction, leading to a partial gasification and rearrangement of the carbon atoms. Due to the inhomogeneous distribution of the Fe_2_O_3_ catalyst particles (Figure 2a), g-NCS-850 and g-NCS-1000 (Figure 3g,h) show a varying degree of perforation and erosion. Within these spheres, fibrous structures probably consisting of graphitic carbon are formed with a thickness of 7–9 nm, as detected by transmission electron microscopy (TEM). The observed thickness matches very well with the average stacking thickness of the graphite layers *L*_c_ determined by X-ray diffraction measurements. Similar observation was made by Liu et al. for carbon spheres that were synthesized by hydrothermal treatment of a sucrose solution and subsequently graphitized in the presence of nickel-oxide particles. High-resolution TEM (HRTEM) images of the resulting particles showed that the graphite layers are arranged along the longitudinal axis of the fibers [37]. After the acidic washing process, neither XPS nor EDX showed, for g-NCS-850 and g-NCS-1000, Fe or Fe_3_C particles within the spheres, which are commonly found for the Fe-based catalytic graphitization of carbon [38]. Hence, under these conditions acid leaching is sufficient to fully remove the metal catalyst. For both sample series, NCS and g-NCS, the particle diameter decreases compared to the initial diameter of the as-synthesized carbon spheres (NCS-550 = 260 ± 35 nm, NCS-1000 = 240 ± 30 nm; g-NCS-550 = 255 ± 35 nm, g-NCS-1000 = 220 ± 30 nm). This is due to the carbonization and decomposition processes taking place at higher reaction temperatures, together with H_2_ etching as side reaction of the ammonia nitriding [34].

The elemental bulk composition of the NCSs and g-NCSs, determined by CHN analyses (supported by EDX measurements, e.g., absence of Fe), as well as the elemental surface composition and N bonding configurations, determined by XPS measurements, are given in Table 1 and Table 2. As expected the samples are made up of a carbon matrix including O- and H-based functional groups [39]. Subsequent N-doping of the carbon lattice results in multiple nitrogen bonding configurations (Table 2). Possible Fe contaminations of the g-NCS samples originating from the Fe_2_O_3_ graphitization catalyst are below the detection limit of the EDX and XPS measurements. The carbonization process of the carbon spheres involves the decomposition of the functional groups to gases such as CO_2_, H_2_O and CH_4_ [39]. Therefore, the carbon weight fraction of the elemental bulk composition increases constantly with higher reaction temperatures, whereas the hydrogen and oxygen contents decrease. The gasification process leads to a lower residual mass, and explains in part the shrinkage of the carbon spheres, as investigated in more depth in our former work [34]. NCS-550 and g-NCS-550 show a bulk N-content of 1.3 wt % and 1.8 wt %, respectively, and the maximum N content is reached for NCS-700 (4.3 wt %) and g-NCS-700 (3.5 wt %). With higher reaction temperatures, the N content of the NCS samples decreases to ca. 1.0 wt %, and even more for the g-NCS samples with a value of only 0.3 wt %. This development is typical for substitutional N-doping of carbon materials by a post-synthetic heat treatment in ammonia atmosphere (see the review by Daems and co-workers [7]). The direct comparison of the N content of NCS-850 and NCS-1000 with the g-NCS-850 and g-NCS-1000 spheres reveals that substitutional N-doping of the amorphous carbon matrix is easier than that of the graphitic one.

**Table 1:** Elemental bulk composition determined by CHN analyses (supported by SEM/EDX measurements).

sample	elemental bulk composition			
	C / wt %	H / wt %	N / wt %	O / wt %

NCS-550	88.3	2.1	1.3	8.3
NCS-700	89.8	1.0	4.3	4.9
NCS-850	95.1	0.7	1.1	3.1
NCS-1000	94.9	0.4	0.9	3.8
g-NCS-550	89.6	1.9	1.8	6.7
g-NCS-700	91.0	1.0	3.5	4.5
g-NCS-850	96.9	0.2	0.3	2.6
g-NCS-1000	97.0	0.2	0.3	2.5

**Figure 5:**
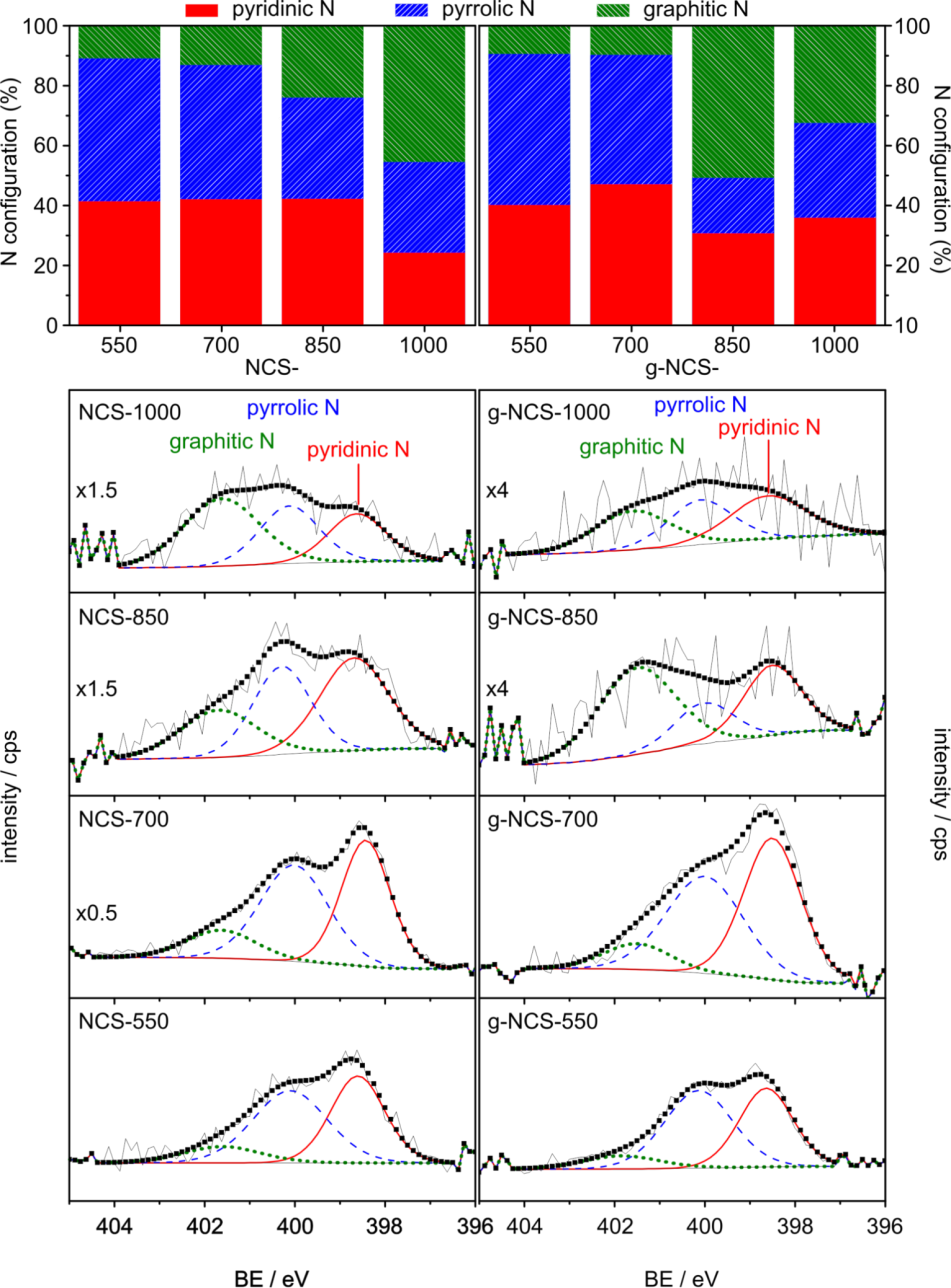
XPS data. Top: N configuration of the NCS and g-NCS catalysts; below: XPS spectra of N 1s region for all catalysts with fitted signals indicating contributions from graphitic, pyrrolic and pyridinic N; the XPS spectra of NCS-1000, NCS-850, NCS-700 and NCS-550 are reprinted with permission from [27], copyright 2019 Elsevier.

The elemental surface composition measured by XPS is similar to the overall elemental composition (CHN analysis), which indicates a homogeneous N-doping of the carbon material. In agreement with the data from elemental analysis, XPS shows the largest amount of N for NCS-700 and g-NCS-700, followed by a strong decrease of the N surface content for (g-)NCS-850 and (g-)NCS-1000. The most plausible bonding configuration of N on the surface is shown in Figure 5. The XPS measurements detect pyridinic N at ca. 398.6 eV, pyrrolic N at ca. 400.1 eV and graphitic N at ca. 401.6 eV on the catalyst surface, whereas no oxidic N could be found at 403–404 eV [15,39–40].

**Table 2:** Elemental surface composition and share of different N bonding configurations determined by XPS measurements.

sample	elemental surface composition			N bonding configuration		
	C / wt %	N / wt %	O / wt %	pyridinic N / wt %	pyrrolic N / wt %	graphitic N / wt %

NCS-550	90.27	0.99	8.74	0.41	0.47	0.11
NCS-700	90.25	2.90	6.85	1.22	1.29	0.38
NCS-850	91.66	0.87	7.47	0.37^a^	0.29^a^	0.21^a^
NCS-1000	92.09	0.59	7.32	0.14^a^	0.18^a^	0.27^a^
g-NCS-550	89.43	1.72	8.85	0.69	0.86	0.16
g-NCS-700	90.57	2.52	6.91	1.18	1.08	0.25
g-NCS-850	95.52	0.34	4.14	0.10^a^	0.06^a^	0.17^a^
g-NCS-1000	95.82	0.29	3.89	0.10^a^	0.09^a^	0.09^a^

^a^Quite large relative deviations are possible for the catalysts nitrided at 850 and 1000 °C due to the low N content yielding a noisy N 1s signal.

NCS spheres that were nitrided at 550 and 700 °C show high fractions of pyridinic and pyrrolic nitrogen atoms. These contents decrease at higher temperatures in favor of an increase of the graphitic N share, reaching around 50% for NCS-1000. A similar trend is found for the N-doped/nitrided graphitized carbon spheres, which show a higher content of graphitic N for the samples treated at 850 °C and 1000 °C, while mainly the amount of pyrrolic N decreases slightly. Here we want to mention that the N 1s signal of the catalysts nitrided at high temperatures is rather noisy, which results in a larger error when evaluating the quantitative amounts of each N configuration. This does not change, however, the trends resulting from the XPS data discussed later.

Focusing on structural aspects, the NCS series, g-NCS-550 and g-NCS-700 samples exhibit XRD patterns characterized by very broad reflections at 2θ values of around 22.5° and 43°, which is typical for amorphous carbon (Figure 4). Obviously, the minimum temperature required for the catalytic graphitization has not been reached for g-NCS-550 and g-NCS-700. This is different for g-NCS-850 and g-NCS-1000, where successful catalytic graphitization is proven by reflections at 26.16° (interplanar distance: *d*_002_ = 0.340 nm) and 26.27° (*d*_002_ = 0.339 nm), respectively, corresponding to the (002) crystal planes of graphite. Applying the Scherrer equation gives an average stacking thickness of the graphite layers *L*_c_ of 7.6 and 8.6 nm, respectively, which matches very well to the thickness of the carbon fibers as detected in the TEM images. The degree of graphitization, *g*, is calculated using the interplanar distance *d*_002_: *g* = (0.344 nm – *d*_002_)/(0.344 nm – 0.3354 nm), with 0.344 nm for the interplanar distance in carbon with a turbostratic structure, and 0.3354 nm for the interplanar distance in a defect-free single crystal of graphite [41–42]. For g-NCS-850 and g-NCS-1000, *g* values of 0.43 and 0.59 were calculated, respectively. The reflections at 41.2° and 43.6° are associated to the (100) and (101) crystal planes of the graphite lattice.

**Figure 6:**
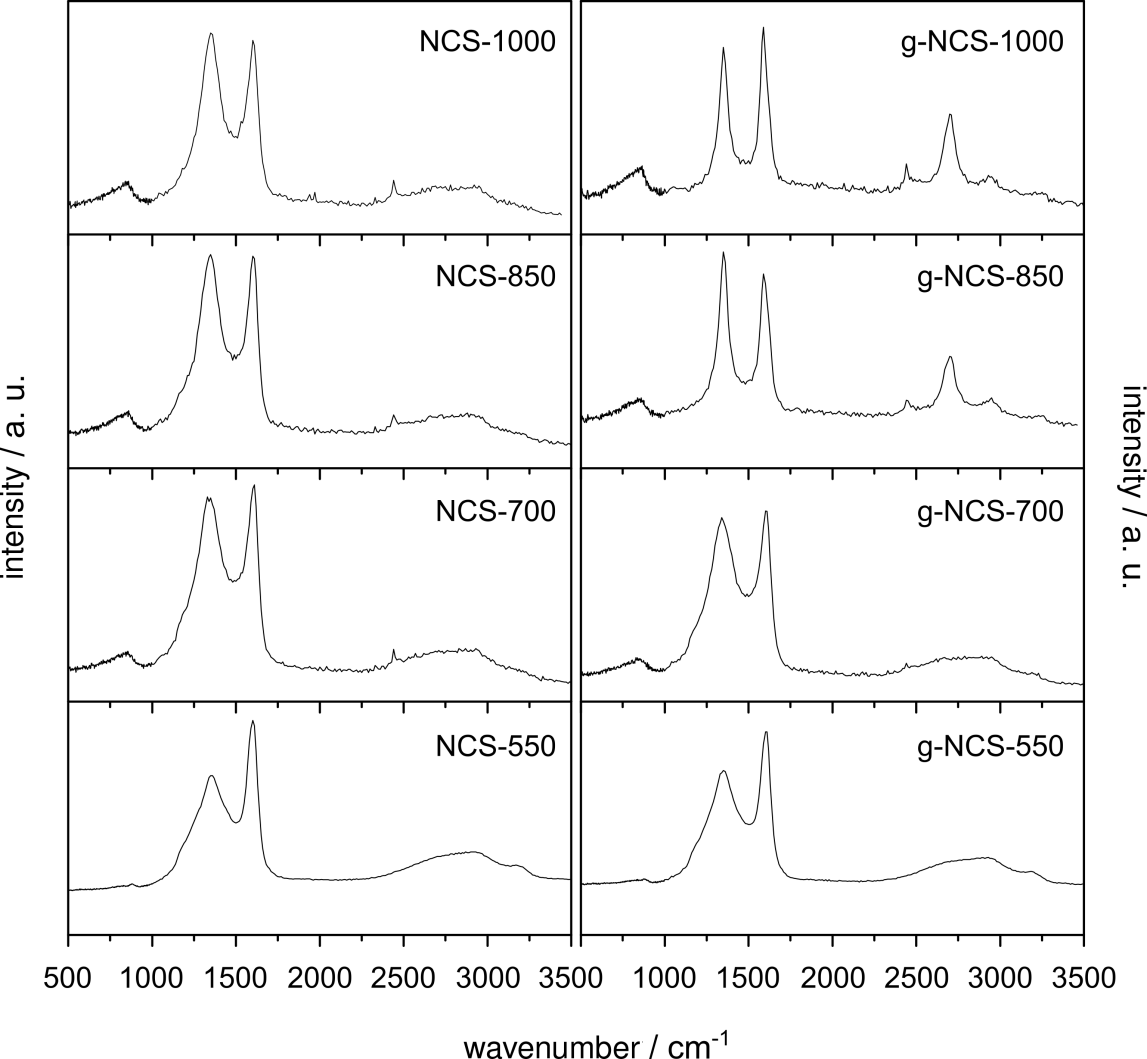
Raman spectra of the NCS and g-NCS catalysts (the *x*-axis represents the Raman shift relative to the excitation laser wavelength given in cm^−1^).

All Raman spectra (Figure 6 and Table 3) of the N-doped carbon spheres show two bands at ca. 1350 cm^−1^ (D band) and ca. 1600 cm^−1^ (G band). The G band is due to the E_2g_ in-plane vibration mode of the graphite lattice and hence assigned to the sp^2^-hybridized carbon atoms inside the graphite layers; the D band is associated to the A_1g_ in-plane breathing vibration mode occurring at the edges of sp^2^-hybridized carbon domains, which appear for structural defects and disordered structures. A relative degree of graphitization can be evaluated by the ratio between the band areas, *A*_D_/*A*_G_; the higher the ratio, the more disordered the carbon material [43–45]. We assume that with higher reaction temperatures the amorphous NCS samples become more ordered through rearrangement to turbostratic-type carbon, indicated by a declining *A*_D_/*A*_G_ ratio (2.7 to 1.9) and a simultaneously decreasing full width at half maximum (FWHM) of the D band. As a result of the structural change from amorphous (disordered) to more graphitic carbon, the *A*_D_/*A*_G_ ratio of g-NCS-850 (1.2) and g-NCS-1000 (1.0) as well as the FWHM of the D band drop significantly. In addition, the D* band (also named 2D or G′ band) at ca. 2700 cm^−1^ is observed as an overtone of the D band, which has the shape we observe for g-NCS-850 and g-NCS-1000, with a shoulder at around 2680 cm^−1^, typically obtained for ordered and disordered graphite [46–47]. The assignment of the low-intensity bands at, e.g., ca. 860 cm^−1^ and ca. 2440 cm^−1^ is described in detail by Kawashima and Dresselhaus and co-workers [46,48]. Higher N contents result in more defects of the carbon lattice and lead to an increase of the *A*_D_/*A*_G_ ratio. Accordingly, NCS-700 and g-NCS-700, which exhibit the highest N content, show the highest *A*_D_/*A*_G_ ratios. For (amorphous) non-doped carbon the G band is located at ca. 1575 cm^−1^. N-doping shifts the G band to higher wavenumbers [49]. This is seen, e.g., for the NCS samples as well for g-NCS-550 and g-NCS-700 with a position of the G band at ca. 1600 cm^−1^. As the N content lowers for g-NCS-850 and g-NCS-1000 the G band shifts back to 1586 cm^−1^ and 1581 cm^−1^, respectively.

**Table 3:** Position and ratio of the band areas *A*_D_/*A*_G_ and full width at half maximum of the carbon D- and G-bands in the Raman spectra.

sample	position (D) / cm^−1^	position (G) / cm^−1^	A_D_/A_G_	FWHM (D) / cm^−1^	FWHM (G) / cm^−1^

NCS-550	1348	1598	2.67	303	78
NCS-700	1338	1602	2.75	267	92
NCS-850	1347	1601	2.54	235	90
NCS-1000	1349	1601	1.87	188	102
g-NCS-550	1345	1600	2.63	285	80
g-NCS-700	1339	1601	2.76	266	92
g-NCS-850	1345	1586	1.24	104	85
g-NCS-1000	1347	1581	1.04	89	71

**Figure 7:**
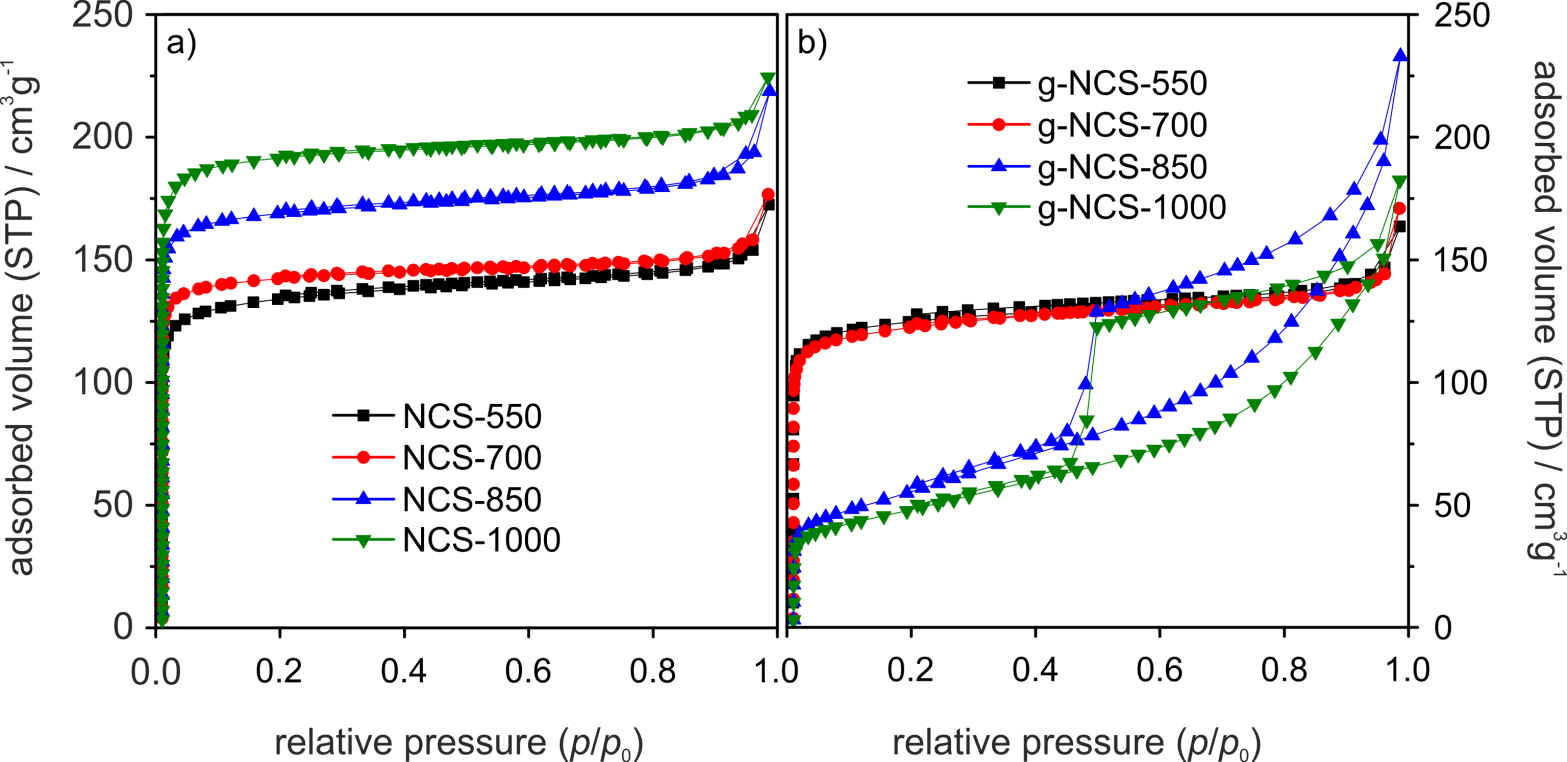
N_2_ sorption isotherms of the (a) NCS and (b) g-NCS catalyst series.

The NCS samples are highly microporous, which is indicated by the measured type-I N_2_ sorption isotherms (Figure 7), combined with a low external surface area compared to the specific surface area (Table 4). The micropore surface area increases with higher reaction temperatures from 485 to 742 m^2^·g^−1^, whereas the external surface area is relatively constant (34–42 m^2^·g^−1^), leading to specific surface areas of 527 m^2^·g^−1^ for NCS-550 to 776 m^2^·g^−1^ for NCS-1000. The formation of micropores is mainly caused by the loss of oxygen, hydrogen and carbon atoms due to gasification and the arrangement to turbostratic-type carbon after heat treatment, as described in more detail in our former publication [36]. The g-NCS samples of lower reaction temperatures (g-NCS-550 and g-NCS-700) are very similar to their NCS counterparts regarding porosity and surface areas. With the onset of graphitization, however, the g-NCS-850 and g-NCS-1000 samples develop a distinct mesoporosity (type-IV isotherms and a H2 hysteresis loop, Figure 7), concomitant with a loss of microporosity of about 66%. The formation of mesopores can be explained by the perforation and erosion of the graphitized N-doped carbon spheres (Figure 2). The micropore system, located within the amorphous carbon, is partially lost with the rearrangement to a more ordered graphitic structure of the carbon lattice. The drastic decrease of the micropore surface area leads to lower specific surface areas of 206 m^2^·g^−1^ for g-NCS-850 and 182 m^2^·g^−1^ for g-NCS-1000 compared to the non-graphitized analogues, NCS-850 (682 m^2^·g^−1^) and NCS-1000 (776 m^2^·g^−1^) (Table 4). For both sample series, NCS and g-NCS, an increase of the adsorbed volume is observed for higher *p*/*p*_0_ values, which can be correlated to the presence of interstitial macropores between agglomerated spheres.

**Table 4:** Specific surface area (SSA), external surface area (ESA), micropore surface area (MPSA) and micropore volume (MPV) measured via N_2_ sorption [SSA = ESA + MPSA].

sample	N_2_ sorption			
	SSA / m^2^·g^−1^	ESA / m^2^·g^−1^	MPSA / m^2^·g^−1^	MPV / cm^3^·g^−1^

NCS-550	527	42	485	0.19
NCS-700	575	39	536	0.21
NCS-850	682	38	644	0.25
NCS-1000	776	34	742	0.28
g-NCS-550	503	39	464	0.18
g-NCS-700	493	50	443	0.17
g-NCS-850	206	168	38	0.02
g-NCS-1000	182	129	53	0.02

In summary, all N-doped carbon spheres of the NCS series, g-NCS-550, and g-NCS-700 are amorphous presumably with only local graphenic structures. A structural change to graphitic carbon is observed for g-NCS-850 and g-NCS-1000. Amorphous carbon spheres show a smooth surface and distinct microporosity; upon graphitization the surface becomes partially perforated or eroded creating a mesoporous system. For both sample series, NCS and g-NCS, the C content increases while the amount of O and H decreases with higher reaction temperatures. The N content has its maximum for (g)-NCS-700; graphitic carbon spheres reveal a lower N content than their amorphous equivalents. Pyridinic, pyrrolic and graphitic N bonding configurations are observed for all samples; here the percentage of the latter increases with higher reaction temperatures.

### 2 Electrochemical and electrocatalytic results

**Figure 8:**
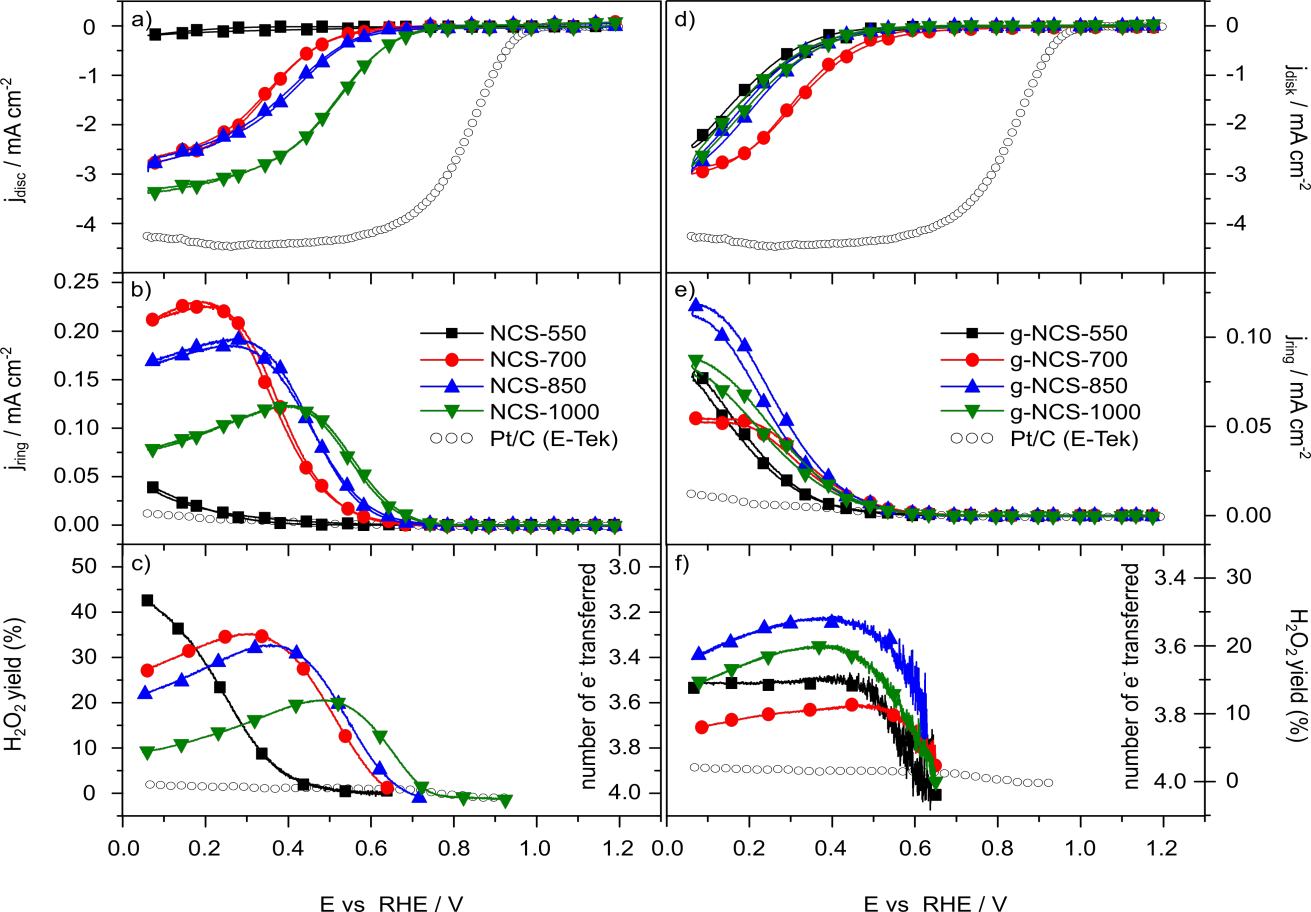
ORR measurements (cyclic voltammograms). (a) ORR disc current densities of the NCS catalysts and Pt/C, (b) ring current densities of the NCS catalysts, (c) hydrogen peroxide yield of the NCS catalysts, (d) ORR disc current densities of the g-NCS catalysts and Pt/C, (e) ring current densities of the g-NCS catalysts and (f) hydrogen peroxide yield of the g-NCS catalysts, 1600 rpm, 0.5 M H_2_SO_4_, 10 mV·s^−1^.

The electrocatalytic ORR activities of the amorphous and graphitized N-doped carbon materials in acidic electrolyte (0.5 M H_2_SO_4_) are compiled in Figure 8, showing (Figure 8a,d) the ORR current densities, (Figure 8b,e) the ring current densities and (Figure 8c,f) the hydrogen peroxide yield. First of all, the data indicate that the carbon NCS-550 spheres are essentially inactive, while with higher nitriding temperatures the NCS samples are significantly more active. For the NCS-550 sample, this inactivity is at least partly due to its high electric resistance determined in resistance measurements (Table 5). For all catalysts nitrided at temperatures above 550 °C, which show a rather low electric resistance (Table 5), conductivity effects can be neglected. We had seen earlier that the trends with increasing nitridation temperature for the ORR in acidic and alkaline media are identical and only the overpotentials are lower in the latter case. Therefore, we focussed in this study on acidic electrolytes.

**Table 5:** Resistance of the catalyst films of NCS and g-NCS (without catalyst film: 2 Ω).

sample	550	700	850	1000

NCS	3·10^6^ ± 1·10^4^ Ω	12 ± 4 Ω	5 ± 2 Ω	3 ± 2 Ω
g-NCS	3·10^4^ ± 4·10^3^ Ω	22 ± 5 Ω	7 ± 4 Ω	5 ± 2 Ω

Going to higher nitriding temperatures the onset potential (the potential at 0.1 mA·cm^−2^; Table 6) increases with temperature. The most active sample, the NCS-1000 sample, shows an onset potential of about 0.75 V, which is, however, still more than 200 mV below that of the commercial Pt/C catalyst. Another important aspect of the N-doped carbon spheres is that the current increase with overpotential is much slower than for the Pt/C catalyst. These catalysts do not reach the transport-limited current indicated by the Pt/C catalyst; in fact, they do not seem to reach a constant current at all, indicating that kinetic limitations are active up to very high overpotentials.

**Table 6:** ORR onset potentials (potential value at 0.1 mA·cm^−2^) of the NCS and g-NCS catalysts in the ORR measurements in Figure 8.

sample	ORR onset potential / V

NCS-550	0.50
NCS-700	0.65
NCS-850	0.70
NCS-1000	0.75
g-NCS-550	0.55
g-NCS-700	0.65
g-NCS-850	0.50
g-NCS-1000	0.50

**Figure 9:**
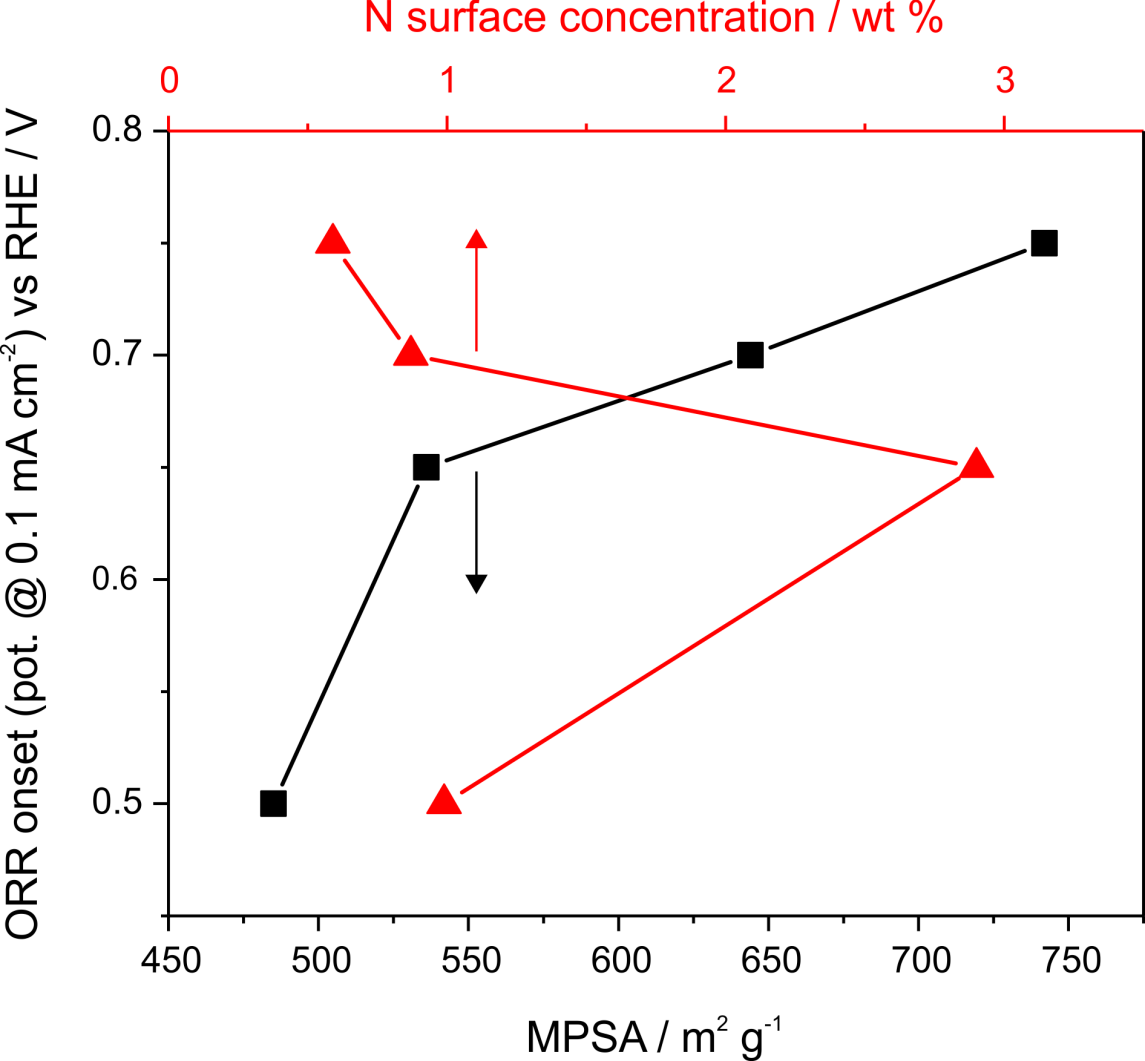
ORR onset potential (potential value at 0.1 mA·cm^−2^) as a function of the micropore surface area (MPSA) and N surface concentration (XPS) for the NCS catalysts.

Correlating the trend in the ORR activity, as indicated by the ORR onset potential (Table 6) and the current density, with the N content of the surfaces (Table 2) for the non-graphitized catalysts, we would expect the highest ORR activity for NCS-700, since here the amount of surface N is largest for each bonding configuration. The data in Figure 8a show, however, a different trend, with the ORR activities of the NCS catalysts growing with increasing nitriding temperature. Hence, there is no direct correlation between the N surface content and the ORR activity of the catalysts, as shown in Figure 9. However, with increasing nitriding temperature, the microporosity increases strongly (Figure 9) and additionally we found a slight increase of the amount of graphenic structures in the catalysts, as indicated by the peak narrowing in the XRD patterns and the decreasing *A*_D_/*A*_G_ ratio in the Raman signals. These structural changes may explain the increase of the ORR activity with higher nitriding temperatures, since previous calculations indicated that the ORR activity of the nitrided carbon catalysts results from the carbon edge atoms of micropores in low-level N-doped (graphitic and pyridinic N) graphene structures [24,26]. Thus, higher nitriding temperatures result in an increase of the proposed ORR active structures for the NCS catalysts. The amount of pyridinic sites, which is often correlated with the ORR activity of nitrided carbon materials [16,22–25], is highest for the NCS-700 catalyst and decreases with higher nitriding temperatures. This is opposite to the trend of the ORR activity, which increases with nitriding temperature (Table 2). Similar, also the concentration of pyrrolic nitrogen sites decreases with increasing nitridation temperature, while the amount of graphitic N, which is sometimes also reported to be correlated with the ORR activity [17–21], is only slightly lower for nitriding at 1000 °C compared to nitriding at 700 °C. Hence, none of these different nitrogen configurations can simply explain the trend in the ORR activity. This agrees with the results of DFT-based calculations of a comparable model system, which showed that the active sites are not the N-sites themselves but rather carbon atoms at edge sites of pores in N-doped graphenic layers [26]. These calculations showed that too high amounts of N-doping and thus of graphitic N-sites can impair the ORR activity, in agreement with our observation that there is no simple correlation between the concentration of graphenic sites and the ORR activity. Instead, we suggest that for the catalysts presented here the changes in the ORR activity are mainly caused by the structural changes, in particular by the microporosity, which increases drastically with increasing nitriding temperature, rather than by the changes in the content of specific nitrogen configurations.

Moving on to the graphitized g-NCS catalysts, the trend for the ORR activities is different (Figure 8, Table 6). For the graphitized samples, the differences between the ORR activities at different nitriding temperatures are significantly smaller. The g-NCS-550 catalyst also suffers from a high ohmic resistance of the catalyst film, which, however, is two decades lower than that of NCS-550. Accordingly, the g-NCS-550 sample is significantly more active than the NCS-550 catalyst. The g-NCS-700 and NCS-700 samples show about the same ORR activity, and for nitriding temperatures above 700 °C, the ORR activities are lower again and clearly below those of the corresponding NCS samples.

The higher ORR current for the g-NCS-550 catalyst compared to NCS-550 can be caused by the higher N content of the g-NCS-550 catalyst, but also by the lower ohmic resistance of the catalyst film (Table 5). Since the g-NCS-700 catalyst shows no graphitization of the carbon (see section 1 in “Results and Discussion”) and also otherwise closely resembles the NCS-700 material (similar N-configuration, N content, SSA/ESA, and microporosity), it is not astonishing that these two materials show comparable ORR activities (Figure 8a,d). Additionally, this result also strongly supports our claim that the acidic washing of the graphitized catalysts is able to largely remove the iron, since otherwise one would expect an increased ORR activity of the g-NCS-700 material. Similar to the NCS-850 and NCS-1000 samples, also for the g-NCS-850 and g-NCS-1000 catalysts the N content decreases significantly for every N configuration with higher nitriding temperatures. The decrease is, however, more pronounced for the graphitized samples. Our previous suggestion that the ORR activity is related to carbon edge atoms at micropore structures in low-level N-doped graphene structures [24] can explain the decrease of the ORR activity of the g-NCS-850 and g-NCS-1000 samples compared with the non-graphitized counterparts, since the amount of micropores is drastically lower after graphitization (g-NCS-850 and g-NCS-1000). Overall, it seems that at higher temperatures (850 °C and above) the graphitization process has a negative impact on the ORR activity of the carbon spheres because of the decrease of the number of micropores, and thus of ORR active defect sites.

Finally, considering the selectivity for the 4-electron reaction pathway to H_2_O, which is highly important for technical applications (Figure 8), we find that at potentials below 0.6 V the NCS-1000 catalyst, the best ORR catalyst in this series, has H_2_O_2_ yields between 10% and 20%, whereas the other catalysts show values between 20% and 40%. Regardless of that difference, the values are several times higher than the H_2_O_2_ yields obtained for commercial Pt-based catalysts (Figure 8). Hence, for technical applications in conventional PEMFCs, it is not only necessary to further improve the activity of nitrided carbon catalysts, but in particular also the selectivity for H_2_O formation. The trend of higher H_2_O_2_ yields at higher overpotentials furthermore clearly demonstrates that the slow increase of the measured current densities towards the transport limited current cannot result from a transition from a 2-electron pathway to a 4-electron pathway with increasing overpotential.

**Figure 10:**
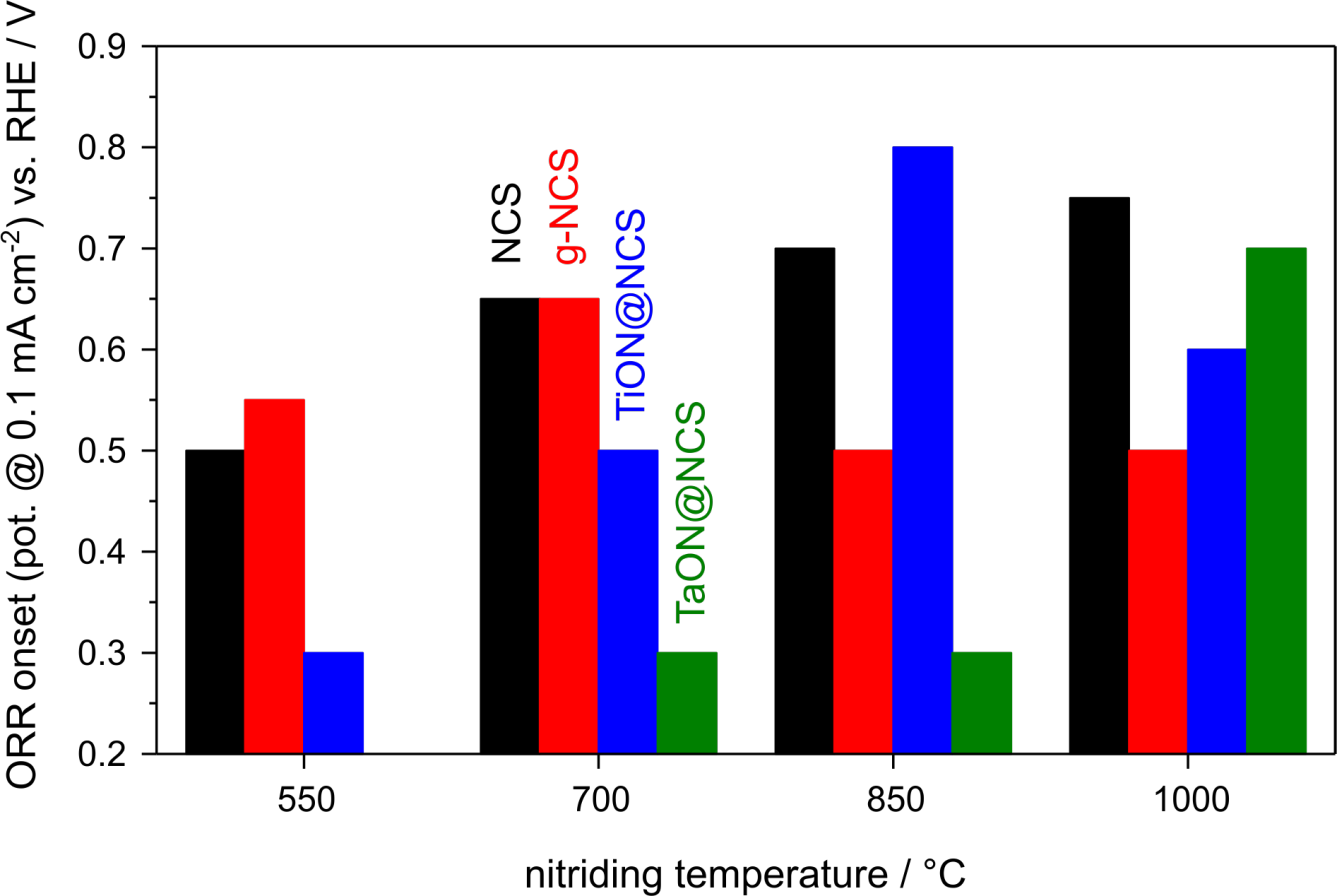
ORR onset potential (potential value at 0.1 mA·cm^−2^) as a function of the nitriding temperature of NCS, g-NCS, TiON@NCS [33] and TaON@NCS [35].

The N-doped carbon spheres investigated in the present study were previously used as conducting carbon cores for composite catalysts (summary of the results in Figure 10), where they were covered by a layer of N-doped TiO_2_ (TiON@NCS) [34] or N-doped Ta*_x_*O*_y_* (TaON@NCS) [33]. The covering was supposed to serve two purposes, first, to yield ORR activity and, second, to protect the (nitrided) carbon core against corrosion. In this study we used the same procedure for N-doping as applied in the present work, which resulted not only in doping of the oxide shell, but also of the carbon core. It was not clear, whether the significant ORR activity of these composite materials is due to the oxynitride shell or perhaps predominantly caused by the nitrided carbon cores. Further insight shall be given by the present study. However, one has to keep in mind, that a quantitative comparison will not be possible. We do not expect nitridation to give the exact same results with and without the presence of an oxide shell due to accessibility and diffusion limitations.

For TiON@NCS [34] we found, in general, similar ORR characteristics as in the present study, with a significantly lower slope of the kinetic ORR current densities in the onset potential range than for Pt/C. In addition, a purely transport-limited region was not reached. In this case the most active sample was that obtained upon nitriding at 850 °C, with an ORR onset at about 0.8 V, in contrast to the best NCS-1000 catalyst, for which an only slighly lower ORR activity was found after nitriding at 1000 °C (Figure 10). Nitriding at 1000 °C led to lower activities for the composite materials, with an ORR onset at about 0.6 V. For the TiON@NCS samples this can be explained by the structural development of the oxynitride shell upon nitridation. For TiON@NCS-1000 the SSA and MPSA values were lower than for TiON@NCS-850; for the latter one a pronounced mesopore formation of the TiON shell resulted in a better accessibility of the N-doped carbon core in the electrochemical studies, which also resulted in a better performance. For the NCS samples only the microporous character becomes more pronounced with increasing nitriding temperature. Hence, the ORR performance cannot be assumed to be identical in both cases, even if it were dominated only by the carbon core. Therefore, a simple quantification of the effect of the shell on the ORR performance is not possible. Nevertheless, it is clear from this comparison that the oxynitride shell does not lead to a general improvement of the ORR performance of the nitrided carbon spheres. This is true also for the H_2_O_2_ yields, which tend to be similar if not higher on the TiON@NCS composite catalysts (20–40%) than on the nitrided carbon spheres.

A similar comparison with the TaON@NCS catalyst [33] shows even more distinct differences (Figure 10). For these composite materials we found a clear ORR activity only after nitriding at 1000 °C. Only the sample TaON@NCS-1000 exhibited a mesoporous shell, thus improving access to the N-doped carbon core. The resulting TaON@NCS-1000 catalyst features a rather similar onset (0.7 V) as the NCS-1000 sample, and similar *j*–*E* characteristics, but somewhat lower current densities. However, for these composites the differences in porosity/surface area between the oxynitride covered spheres and the pure nitrided carbon spheres are even more pronounced, with substantially lower surface areas for the TaON@NCS catalysts. Hence, in these cases direct comparison of the ORR performance after similar nitriding temperatures is even less possible. Nevertheless, for nitriding temperatures above and below 1000 °C, the TaON layer seems to block the ORR activity of the nitrided carbon cores very likely due to the lack of the permeable mesoporous shell. Also when comparing the H_2_O_2_ yields of the TaON@NCS composites [33], we find no advantage of the composite catalysts since the hydrogen peroxide yields are around 40% for all catalysts, which is higher than the values obtained for the pure nitrided carbon spheres (mostly around 20%).

Overall, the present findings underline that the metal (oxy)nitride shell of the composite catalysts does not lead to a general improvement of their ORR performance. Within the present series of catalysts, the non-graphitized carbon spheres nitrided at 1000 °C are the most suitable Pt-free ORR catalysts. Further work is needed, however, to improve the relative high peroxide yields obtained so far.

## Conclusion

The N-doped carbon spheres (NCS) synthesized in the present work are characterized by a well-defined spherical shape and smooth surface. Originating from glucose, the carbon matrix of the spheres initially contains oxygen- and hydrogen-based functional groups. The N content (pyridinic, pyrrolic and graphitic bonding configurations) has its maximum after nitriding at 700 °C. The carbon structure is amorphous as proven by XRD and TEM measurements, with an increasing tendency to turbostratic-type carbon with higher reaction temperatures.

Graphitized carbon spheres were synthesized with the aid of an iron oxide catalyst at the respective nitriding temperature. For g-NCS-550 and g-NCS-700 materials, the minimum temperature required for the catalytic graphitization is not reached yet, therefore their properties are almost equal to those of the amorphous NCS counterparts. Graphitization at higher temperatures leads to the formation of mesopores, combined with the loss of the micropore system. Within the spheres clew-like strings are observed, their thickness matches the average stacking thickness of the graphite layers *L*_c_ leading to the conclusion, that the graphite layers are arranged along the longitudinal axis of the strings. The N content of the g-NCS catalysts is lower compared to the NCS samples. This ultimately results in a less efficient substitutional N-doping for graphitized carbon spheres.

The nitrided amorphous carbon spheres show a high ORR activity when nitrided at high temperatures (1000 °C), which, however, resulted in the lowest N content for all three N configurations of all NCS catalysts. We attribute the high ORR activity of this catalyst to the large amount of micropores (ORR-active C edge atoms) in low-level N-doped graphenic structures. The graphitization (g-NCS) seems to hinder the ORR activity even after high nitriding temperatures, because of the strong decrease of the micropores compared to the non-graphitized catalysts. In that picture the ORR activity is not associated directly to one of the N sites, but strongly depends on the amount of defect sites and thus on the microporosity/graphitization of the carbon surface, in combination with a low N-doping. These correlations between structure and ORR activity can be used to further improve the catalytic activity of N-doped carbon catalysts towards the ORR.

## Experimental

### Chemicals

All chemicals were purchased from commercial suppliers and were used without further purification unless stated otherwise: Glucose (Amresco, 98%), ethanol (VWR, 99.5%), iron(III) nitrate anhydrous (Sigma-Aldrich, 99.9%), hydrochloric acid (Merck Emsure, 37%), argon (Air Liquide, 99.99%), and ammonia (Air Liquide, 99.9%).

### Synthesis of (graphitized) N-doped carbon spheres

The synthesis of the nitrided carbon spheres (NCS) was analogous to the procedure in our previous publications [27,34]. Carbon spheres were synthesized by hydrothermal treatment of a 0.75 M glucose solution in aqua dest. (165 mL) at 165 °C for 10.5 h. The resulting spherical carbon particles were washed three times with 200 mL aqua dest. and ethanol each, centrifuged and dried [36].

**Synthesis of N-doped carbon spheres (NCS):** The as-synthesized carbon spheres were carbonized under argon atmosphere in a tube furnace (*V* = 12 L) for 4 h (heating rate 5 °C·min^−1^) at different temperatures, between 550 and 1000 °C with steps of 150 °C, followed by N-doping in an ammonia atmosphere (3 NL·h^−1^), holding the individual carbonization temperature of each sample for 1 h. Cooling to room temperature was performed in an argon flow.

**Synthesis of graphitized N-doped carbon spheres (g-NCS):** As-synthesized carbon spheres were pre-carbonized in argon atmosphere for 1 h (heating rate 5 °C·min^−1^) at 550 °C. A solution of 5.05 g iron(III) nitrate in 50 mL aqua dest. was added to 2.5 g pre-carbonized carbon spheres and stirred for 24 h, followed by refluxing for 5 h at 100 °C and subsequent filtration and drying. Catalytic graphitization was carried out by annealing at different temperatures, between 550 and 1000 °C with steps of 150 °C, in argon atmosphere for 4 h (heating rate 5 °C·min^−1^). Iron catalyst particles were removed by acid leaching with 2 M hydrochloric acid, followed by filtration, washing with aqua dest. to a neutral pH value and drying [32]. N-doping was realized in the same way as described for the NCS sample series; thereby the N-doping temperature is set to the same temperature as used for the catalytic graphitization of the given sample.

In the following, N-doped carbon spheres are labeled as NCS and graphitized N-doped carbon spheres as g-NCS. The number added to those labels represents the reaction temperature.

### Characterization of (graphitized) N-doped carbon spheres

Scanning electron microscopy (SEM) images were recorded with a field-emission scanning electron microscope (FE-SEM, Zeiss Ultra Plus) at 10 to 12 keV beam energy. For imaging, the samples were deposited on a conducting carbon film. Energy-dispersive X-ray spectroscopy (EDX) measurements were performed on the same FE-SEM with an EDX large-area silicon-drift detector (Oxford X-Max 50), using an accelerating voltage of 15 kV with a counting time of 5 min per spot. Bright-field transmission electron microscopy (BF-TEM) images were taken with a JEOL1400 instrument equipped with a CCD camera. For sample preparation, a droplet of ethanol containing the dispersed sample powder (ca. 1 mg·mL^−1^) was deposited on a carbonized Cu grid (Plano, Mesh 300), followed by evaporation of ethanol. For CHN elemental analysis, a Vario MICRO cube instrument (Elementar Analysensysteme GmbH) was used, the thermal decomposition temperature was 1000 °C in air. XPS measurements were performed in a Physical Electronics PHI 5800 Multi ESCA system at an emission angle of 45° and a pass energy of 29.35 eV (detail spectra), applying monochromatic Al Kα radiation (250 W, 13 kV). The thin-layer samples used for these measurements were prepared by depositing and drying 20 μL of an aqueous catalyst suspension on a silicon wafer, which was pre-cleaned by sequential rinsing in ultrapure water (MilliQ), 1 M KOH solution, and conc. H_2_SO_4_. By using silicon wafers instead of a carbon-containing support, we minimized contributions from the support to the C 1s signal of the carbon-containing catalyst film. The spectra showed minor charging effects, which were compensated by a neutralizer (low-energy electron flood gun). The C 1s peak was set to 284.8 eV for binding energy calibration [50]. Evaluation and deconvolution of the measured signals (Shirley background; peak shape: 70% Gaussian/30% Lorentzian) was carried out using the CasaXPS software package. X-ray diffraction (XRD) measurements were performed using a Bruker D8 Advance instrument (Bruker Karlsruhe), employing Cu Kα radiation (λ = 0.154 nm) in a 2θ range of 5° to 80° (0.02° continuous mode, 0.5 s per step). Porosity and specific surface area were determined by N_2_ sorption measurements on a Micromeritics ASAP 2420 instrument (Micromeritics) in a relative pressure range of *p*/*p*_0_ between 4 × 10^−6^ and 0.99 and a temperature of −196 °C. The specific surface area was calculated by the method of Brunauer, Emmett, and Teller in a relative pressure range of *p*/*p*_0_ 0.01 to 0.3. The ratio of micropore surface area to external surface area was calculated by the t-plot method (thickness curve: carbon black STSA, fitted thickness range: 0.4–0.6 nm). Raman spectroscopy measurements were performed by a Thermo DXR Raman microscope (Thermo, Madison) with a confocal microscope BX41 (Olympus Corp.). The diameter of the laser spot was approximately 2.5 µm (10× microscope objective, NA = 0.25), the laser power was 1 mW at 532 nm, the spectra were collected from 100 to 3700 cm^−1^ with a spectral resolution of 5 cm^−1^ (50 µm slit-like pinhole) with an exposure time of 5 s (10 accumulations).

### Electrode preparation and electrochemical measurements

The catalyst thin-film electrode (catalyst loading of 0.285 mg·cm^−2^ for Pt-free catalyst, 140 μg·cm^−2^ loading (Pt loading: 28 μg·cm^−2^) for the 20 wt % Pt/C E-Tek reference catalyst) was prepared by pipetting an aqueous suspension of the synthesized materials (20 µL of a 4 mg·mL^−1^ suspension; Millipore MilliQ, 18.2 MΩ·cm) onto a mirror-polished glassy carbon (GC) disc (Sigradur G from Hochtemperatur Werkstoffe, *d* = 6 mm), followed by subsequent drying under a N_2_ stream. With these loadings we could form homogeneous, thin and stable catalyst layers on the electrode. The resulting film was covered with the same volume of a 1 wt % aqueous Nafion solution and dried again to ensure the mechanical stability of the catalyst layer on the glassy carbon without creating additional diffusion limitations [51]. The geometric area of the electrochemically accessible part of the electrode is 0.28 cm^2^. For the electrochemical experiments, we used a rotating ring disk electrode (RRDE) setup (Pine Instruments Analytical Rotator, AFASRE), with the thin-film electrode on the GC disc functioning as working electrode. The working electrode is surrounded by a Pt ring biased at 1.2 V, which allows one to measure the peroxide yield in the ORR. A reversible hydrogen electrode (RHE) served as reference electrode and a Pt wire as counter electrode, both separated by glass frits from the main cell. The RHE itself consists of a Pt plate in a glass tube containing the respective electrolyte used for the measurement and a H_2_ bubbler. In the following, all potentials will be quoted versus that of the RHE. The potential was controlled by a bi-potentiostat (Pine Instruments AFRDE5). The potentiodynamic ORR measurements were performed in acidic electrolyte (0.5 M H_2_SO_4,_ Merck Suprapur, Millipore MilliQ, 18.2 MΩ·cm) in O_2_ saturated supporting electrolyte at a scan rate of 10 mV·s^−1^ and a rotation rate of 1600 rpm. For all ORR measurements, the currents in N_2_-saturated electrolyte were subtracted from the measured ORR currents in order to remove double-layer charging currents. For each catalyst the cyclic voltammograms are presented, thus the ORR measurements of each catalyst consist of a cathodic (down-going scan, lower trace) and an anodic (up-going scan, upper trace) scan. For the calculation of the hydrogen peroxide yield we used Equation 1, where *I*_r_ is the measured ring current, *I*_d_ the disc current and *N* the collection efficiency of the setup (here the measured value specific for the current setup of N is 0.2):

[1] $${\text{H}}_{\text{2}}{\text{O}}_{\text{2}}\left(\%\right)=\frac{2\left|I_{\text{r}}\right|}{N\left|I_{\text{d}}\right|+\left|I_{\text{r}}\right|}\cdot 100.$$

The resistance measurements of the catalyst film were performed by pipetting and drying 80 µL of the catalysts suspension on a glassy carbon disk, similar to preparation of the catalyst film for the electrochemical measurements. The dried catalyst film is than covered by another glassy carbon disk, and the two disks were tightly pressed together. The resistance between both glassy carbon units with the catalyst film in between was measured with a Keithley 197A multimeter. The onset potentials in Table 6 and Figure 9 and Figure 10 are defined as the potential at which the current geometric densities exceed 0.1 mA·cm^−2^.
